# Dr. Hamilton’s Surgical Clinique, at Geneva Medical College

**Published:** 1847-03

**Authors:** 


### BUFFALO MEDICAL JOURNAL
AND
### MONTHLY REVIEW.

VOL. 2. MARCH, 1847. NO. 10

### ORIGINAL COMMUNICATIONS.

ART. I.—Dr. Hamilton's *Surgical Clinique, at Geneva Medical College. Reported for the Buffalo Medical Journal.*

*Closure of Wharton's Duct.*—C. S. H., aged 24. Right submaxillary gland enlarged to size of a hickory nut. A week since, during breakfast, he first discovered the enlargement; in five minutes it became as large again as it now is, and occasioned considerable inconvenience when he opened his mouth. He immediately began to rub it, and in a short time it was reduced one half. From that day it has swollen up regularly at each meal and been again partially reduced by friction: it is larger during breakfast than any other meal; it will not diminish without rubbing; does not matter what kind of food he eats; chewing tobacco rather diminishes it; not sore or painful, except that when he eats, a pain is produced which extends to the ear. Tongue furred, some indigestion, has not felt well the last few days.

It is a case of slight inflammation and temporary closure of the duct of Wharton.

Dr. H. prescribed a full dose of Sulphas Magnesiæ. The next day it had entirely disappeared, and two weeks after it had not returned.

*Obstruction of Duct of Steno.* S. C., aged 28. Some time since a swelling occurred opposite the opening of the duct of Steno, which after a time suppurated and discharged the saliva externally. Soon after it closed and a hard schirrous tumor formed an inch below. This has opened twice and discharged pus. It is evidently connected with the fistula.

The original obstruction was caused by an inflammation produced by a carious tooth: patient very plethoric.

He was advised to submit for a time to a low diet and other preparatives and then resort to an operation for the cure of the fistula.

*Stricture at mouth of Urethra.* A. C. aged 33; from Otisco: deaf and dumb. When quite young he was injured by a playmate and a stricture resulted, extending from the external meatus upward two inches. Two years since Dr. H. operated upon him before the class by puncturing in the direction of the channel and introducing a short silver catheter which was to be kept in until a cure was effected. It was allowed to slip out and was not replaced and the stricture has returned, but not as bad as before. A small metallic *sonde conique* was introduced and his father was directed to repeat the introduction daily, gradually enlarging the instrument until the urethra was sufficiently dilated.

*Strabismus Internus.*—R. P., of Geneva, aged 17. Has internal strabismus of 3d degree. Parents affirm that it is *congenital.* Both eyes squint equally; myoptic; eyes prominent.

Dr. H. cut the internal rectus of left eye; parallelism almost perfectly restored. Two weeks after, she went home with the eyes looking straight and natural.

*Adipose Tumor.*—Miss E. M'N., aged 25. Tumor on back of right shoulder, of eighteen months growth. Occasionally sore and painful, producing stiffness of neck. It was removed by a free incision and closed by straps. She had been advised to try the finger of a *dead man*, but no favorable opportunity had occurred for the experiment.

*Soft polypus of Nose.*—I. T., of Steuben Co., aged 13 years : commenced in left nostril a year since ; can be seen ; is not affected by weather ; child not healthy.

Dr. H. removed with forceps and wire ligature all that could be reached. Left him in care of Dr. Foote, with a recommendation that he apply daily, white precipitate ointment with a camels hair pencil, to superior meatus ; introducing it well back and sweeping the whole roof of the meatus.

*Enlargement of Submaxilary Gland.* J. C. L., aged 20; Yates Co. In latter part of July last, patient first detected in the morning a soreness under left side of tongue. (He had labored the day previous in the harvest field.) Before night he felt an oblong tumor which Dr. W. opened immediately and it discharged a glairy fluid. Four days after, it having closed again, it was re-opened and three times afterwards, at intervals of from ten days to four weeks. This Dr. H. regarded as an obstruction of one of the ducts of the sublingual gland. But finally this obstruction has ceased and the disease is transferred to the submaxillary of the opposite side, which is growing rapidly yet it is not sore or painful. It is much larger in cold, damp weather than in warm or dry,

Patient was recommended not to submit to an operation for its extirpation until he had tried therapeutic means. As he was exceedingly plethoric, he was advised to place himself on a vegetable diet—take cathartics occasionally—and use internally tr. iod. gtta xv three times per day; and apply the ung. hyd. iod. pot. externally.

*Carcinoma.*—Jacob Clapper, of Rose Valley, aged 56. Farmer; healthy; temperate; no hereditary predisposition to cancer; has naturally very large breasts. Seven years since, he discovered a tumour, size of a pea, hard and painless, about an inch below right nipple. A year since, it had attained the size of a hazlenut; had never boen sore or painful, except when roughly handled; skin looked natural, but the tumour was very hard. At this time, it was seen by a Doctor, who applied a plaster, and in two weeks it was an open ulcer. Both the induration ond ulceration now extended rapidly, nor has the ulcer at any time shown the least disposition to heal.

Its present appearance is bad enough—an irregular, jagged, ill-conditioned ulcer, of three inches in diameter, resting upon a much elevated and very hard base, of fonr or five inches in diameter—the whole mass firmly adherent to the muscles and bone underneath.

Dr. H., finding that it had become very much inflamed from exposure during the last few days, directed him to return home and confine himself to a low diet for a few weeks, and when both the part and his general system were properly prepared, the operation of extirpation should be made. He promises to present himself before the class at Buffalo in the spring.

*Melanosis of the Eye.*—Wm. Reid, Irish, aged 46, resides in Nunda. Twenty-three years since, a small, light brown spot appeared at junction of cornea with sclerotica on outside of left eye: it gradually increased, and about twelve years since, having attainee the size of a walnut, it was cut off close to the sclerotica, by Dr. Little, of the Infirmary at Sligo, Ireland. Its colour was then, as now, a dark blue-black. One year since, it was again removed by Dr. Munn of Rochester, who then declared to him that it was malignant, and he ought to submit at once to extirpation of the eye—but Reid refused to take his advice.

It is now size of a marble, projecting between the lids and preventing their closure; its surface is slightly lobulated; its feel, pretty firm; it is not sore or painful. Three-fourths of the cornea is perfectly sound and transparent, but a broad inky streak extends half way around the cornea, along its margin and under the conjunctiva. It is the same melanotic matter which forms the tumour. Patient is in good health.

Reid being told that complete removal of the eye might effectually eradicate the disease, and that, if allowed to remain, it was almost certain to prove fatal ultimately, he consented to the operation. First, however, he requested permission to address the class, feeling, we suppose, like one sentenced to death, and who begs to make his final speech before he bows to the knife of the executioner. His address being made, he stretched himself meekly upon the operating table, and never moved, or sighed, or gave sign of pain until the operation was completed. Neither Morton's "Letheon," nor the most expert mesmerizer, could have more effectually sealed up all evidence of pain.

The operation was made in the usual manner. No vessels required the ligature—no lint was placed in the socket. It was dressed with a linen cloth wet in cold water. Ten days after, the socket was nearly filled with granulations, and the patient returned home, feeling quite well.

*Encysted, Melliceroous Tumor.*—V. Webb, aged 40, from Tioga, Penn. This tumor has been growing three years under left angle of inferior maxilla: now size of a goose-egg.

*Operation.*—A long incision was made over the face of the tumor down to fascia superficialis; the external jugular was then tied, and the incisions continued until the tumor was laid open and its contents discharged. The base of the sack was attached to tho sheath of the internal carotid and internal jugular; its upper part extended, in a long, finger-like projection, up and back between the ramus of the jaw and the mastoid process, and under the sterno-cleido-mastoid muscle. It was not thought advisable to follow this prolongation, all of the balance of the sac having been removed. The superior thyroid artery was tied during the operation. It was dressed by introducing a piece of lint to the base of the wound and partially closing the external wound with sutures and straps. Two weeks after, the patient went home, the wound having nearly closed.

*Sarcoma of integument.*—W. R., of Pennsylvauia, aged 25; had a small hard tumor on the integument of the lower part of the abdomen two or three years since, It was removed and the wound healed in six weeks by granulation. When the cicitrization was completed, a similar, but harder tumor began to form in the corner of the cicatrix and soon extended its entire length. It is now the size of a butternut, very hard; skin hard, tense and iucorporated with tumor; not painful or tender. Patient healthy, no hereditary predisposition to carcinoma.

It was removed by a double elliptical incision, made free from the borders of the tumor and closed by sutures and adhesive straps. Patient having walked nearly a mile to his boarding house, a secondary hæmorrhage occurred which required all the ligatures to be removed. It was then allowed to unite by granulation. It healed kindly.

*Encysted tumor of lips.* M. C., aged 19; from near Geneva; has a tumor of under lip of size of filbert; feels slightly elastic, yet harder than ordinary encysted tumors; it is not moveable under the skin; not painful.

On exposing it for removal it was found firmly adherent to the structure of the lips on all sides; a portion of its interior was hard and organized like a sarcoma; and a portion was soft and mellicerous. One ligature was applied and the wound closed by suture.

*Congenital Hydrocele.* John Ringer, aged 8 years; two miles north of Geneva; has a congenital hydrocele of left side; cylindrical, not pyriform; translucent; contains about five ounces of fluid. As no operation had ever been made and the child was young it was thought proper to simply evacuate the sac; there was a fair chance that it would result in a radical cure. Opened with a bistoury.

*Enlarged Tonsils.*—A. G., aged 12, Ontario, Wayne Co. Enlargement resulted from measles. Hearing much impaired by pressure on Eustachian tube. Dr. H. removed both freely; hæmorrhage very slight.

*Enlarged Tonsils.*—M. G., aged 12, from near Geneva. Produced by Scarlatina; hearing not impaired. Both were removed freely; bled about half an ounce. The sister of the above girl has enlarged tonsils also, but they were too small to require removal.

*Enlarged Tonsils.*—I. Graves, Benton, Yates Co., aged 8 years. Last January Dr. H. excised one; no appearance of gland on that side. To-day removed the other.

*Closed Pupil.*—I. H. H., of Cincinnatus, Cortland Co., aged 23. Had a violent opthalmia a year since; treated without depletion; eye washes alone used for some weeks. Pupil is closed with a membranous deposit; complete immobility of pupil.

*Capsular Cataract.* Opposite eye became affected two or three months afterwards. A capsular cataract is forming,

Dr. H. recommended him to wait until the cataract was complete and then submit to an operation upon one of the eyes: it was not material which.

*Adhesion of iris to cornea; leucoma,* &c. Mrs. D. aged 43, of Branchport. Had acute opthalmia three years ago; was not bled, but took emetics and cathartics. An ulcer resulted in left eye and a protrusion with adhesion of the iris; a leucoma in seat of ulcer; whole ball atrophied. Right eye in nearly same condition. Patient can distinguish daylight from darkness.

A solution of nit. arg. was recommended to remove the remaining passive inflammation in right eye, and an operation for artificial pupil in left.

*Amaurosis from congestion.* R. C. Been subject to headaches, with vertigo, fulness of head &c., during last three years: is habitually costive; has been reading much the last year; has cold feet. He began to see muscæ volitantes nine months since.

Dr. H. recommended vegetable diet, cathartics, seton in back of neck, leeches to temples, loosen neckcloth, keep head cool and feet warm, refrain from reading, take exercise. Prognosis is favorable, if patient follows advice.

*Incipient Cataract.* Mrs. P. H. of Sodus, Wayne Co., aged 50. Had acute opthalmia five years since; sight been failing ever since. Sees a halo about a candle ; sees best in a moderate light; objects often double and confused: has never seen motes; pupil contracts and dilates tolerably; catoptric test presents two images.

*Glaucoma.*—Dr. J—— H——, of Potter, Yates Co., aged 47. Dr. H—— has, for twenty-five years, been accustomed to read after he retired to bed. Two years since, he began to see objects double, and had vertigo, &c.; vision has gradually failed him from that time; had very little pain; no fullness of balls; slight vertigo of late; never saw motes; cannot distinguish forms of bodies. The following circumstances were noted as indicating some opacity of lens: sees best in a moderate light; sees the stars and a candle best when looked at obliquely. But the opacity in the pupil is deep-seated and slightly brown. The catoptric test shows three images, but the third is feeble.

Dr. H. regards it as an opacity of posterior capsule of lens, and amaurosis conjoined possibly with opacity of the vitreous humor; he would call it glaucoma. Treatment, antiphlogistic and counter-irritent.

*Chronic Inflammation of Tarsi.*—Miss J. W. of B., aged 14, has had this inflammation since she was two years old. Lids now badly everted, tumid, and of a bright scarlet colour. Complexion fair and face full; texture scrophulous. A sister had a similar opthalmia, from infancy until eight years old.

Dr. Smith, Oculist of Geneva, presented this case for the instruction of the class. She has been in charge of Dr. S. five weeks: he has been giving her generous diet, and has applied nit. arg. in substance every other day. The improvement has been rapid.

*Leucoma.*—An Irishman presented himself with a leucoma as the result of a burn of the cornea from lime. Incurable.

*Entropion.*—Mrs. N., Ontario Co. Last year, Dr. H. operated on right upper lid, for a ptosis; cure complete. She now wishes an operation for entropion of left lower lid. Dr. H. removed, with scissors, an elliptical piece of integument, of length of lid, and four lines in breadth—and closed wound with four very delicate silk sutures; the sutures were armed with a fine cambric needle.

*Ptosis.*—J. P. of Vienna, aged 43. Had acute opthalmia twenty-five years ago. Both eyelids been falling gradually ever since. Dr. H. operated upon both eyes; removing a portion of each upper lid, of nine lines in breath and of length of lids, with scissors and cross-barred forceps. Closed wounds with five sutures on each lid: used cambric needles again. Lids opened well.

*Otoplasty.* Z. H. B., aged 24. Had his left ear torn off close to head by the kick of a horse, nothing remaining but the lobe, occurred when eight years old. Dr. Cook re-applied it and bound it together. It is now perfectly in place and entire; the cicatrix however remains to confirm the gentleman's statement.

*Deafness.* L. M. K. Seneca Co., aged 34. Deafness commenced in right ear 16 years ago; was then a farmer and in good health. 12 years since had repeated attacks of remittent fever and his constitution became much impaired. Nine years since it commenced in left ear. Can now hear with right ear the tick of a watch two inches and with left, six inches. Has always heard chirping and roaring sounds—sounds like birds, toads, chickens, &c; feels the roaring especially at each pulsation of the artery. A long continued effort to hear increases the deafness very much, and it is again improved by a walk in the cold air: hears better in cold air than warm; never has ear-ache, nor head ache; eustachain tube is pervious. Has been very feeble the last fourteen years.

This is a case of deafness from impairment of function of nerve, and the patient was recommended te take exercise in the open air; employ tonics and live generously, &c.

*Dumbness without Deafness.*—Margaret Sullivan, aged 8 years, daughter of Michael Sullivan, of Geneva. Hearing perfect; tongue not restricted in its motions ; utters simple sounds clearly and distinctly; head large and well formed; countenance intelligent; has always exhibited all the intelligence of other children and more than many of her mates. She can only articulate eight or ten words, such as "die," "Jane," "horse," "go way," "cow," "Mother," the last requiring a rapid motion of the tongue, she articulates distinctly.

Dr. H. regards this as a case of simple lack of attention and effort on the part of the child at an early period of life; the habit of not speaking having been acquired it will require much instruction to overcome it, but it can certainly be done. There is a physician in Albany of eminence who never articulated a word until seven or eight years old.

*Erysipelas of Legs.*—George B., aged 16. Produced by walking in the cold as a pedlar. Dr. H. recommended Salts, and that Velpeau's solution of Sulphate of Copper be applied as a wash, and his limbs kept in a horizontal posture.

*Varus.*—Infant child of W. R., of Penn Yann, aged two years. Varus in both feet, 3d degree. A *bursa* has formed on the right foot, where the foot touches the floor.

Recommended section of tendo achilles in both feet, and application of scarpa- shoe. Parents not ready for the operation.

Eleven cases of Fracture have been presented, and will be reported separately.

P*****, *Reporter for Buffalo Medical Journal.*

